# Acute Respiratory Distress Syndrome Induced by a Swine 2009 H1N1 Variant in Mice

**DOI:** 10.1371/journal.pone.0029347

**Published:** 2012-01-03

**Authors:** Yi Zhang, Honglei Sun, Lihong Fan, Yuan Ma, Yipeng Sun, Juan Pu, Jun Yang, Jian Qiao, Guangpeng Ma, Jinhua Liu

**Affiliations:** 1 Key Laboratory of Animal Epidemiology and Zoonosis, College of Veterinary Medicine, China Agricultural University, Ministry of Agriculture, Beijing, China; 2 China Rural Technology Development Center, Beijing, China; 3 Shandong Animal Disease Control Center, Shandong, China; University of Giessen Lung Center, Germany

## Abstract

**Background:**

Acute respiratory distress syndrome (ARDS) induced by pandemic 2009 H1N1 influenza virus has been widely reported and was considered the main cause of death in critically ill patients with 2009 H1N1 infection. However, no animal model has been developed for ARDS caused by infection with 2009 H1N1 virus. Here, we present a mouse model of ARDS induced by 2009 H1N1 virus.

**Methodology Principal Findings:**

Mice were inoculated with A/swine/Shandong/731/2009 (SD/09), which was a 2009 H1N1 influenza variant with a G222D mutation in the hemagglutinin. Clinical symptoms were recorded every day. Lung injury was assessed by lung water content and histopathological observation. Arterial blood gas, leukocyte count in the bronchial alveolar lavage fluid and blood, virus titers, and cytokine levels in the lung were measured at various times post-inoculation. Mice infected with SD/09 virus showed typical ARDS symptoms characterized by 60% lethality on days 8–10 post-inoculation, highly edematous lungs, inflammatory cellular infiltration, alveolar and interstitial edema, lung hemorrhage, progressive and severe hypoxemia, and elevated levels of proinflammatory cytokines and chemokines.

**Conclusions/Significance:**

These results suggested that we successfully established an ARDS mouse model induced by a virulent 2009 H1N1 variant without previous adaptation, which may be of benefit for evaluating the pathogenesis or therapy of human ARDS caused by 2009 H1N1 virus.

## Introduction

A novel influenza A (H1N1) virus of swine origin emerged among humans in Mexico during the spring of 2009 and rapidly spread worldwide [1]. The pandemic prompted the World Health Organization (WHO) to raise the alert level to the highest rating of six, the pandemic phase, within 2 months [2]. In August 2010, WHO officially declared that the disease was in the post-pandemic period [3]; however, it is still circulating among humans, together with seasonal viruses. Although most influenza cases caused by 2009 H1N1 virus infection typically display mild upper respiratory tract syndrome, some cases progress to severe pneumonia and acute respiratory distress syndrome (ARDS) [4], [5]. Many studies have shown that ARDS caused by 2009 H1N1 virus results in 17.3–56% mortality [4], [6], [7], [8], which was regarded as the major cause of death by 2009 H1N1 virus infection [9]. ARDS is the result of acute injury to lung tissue, commonly resulting from sepsis, trauma, and severe pulmonary infections [10]. Infectious factors, most of which are viruses, have become one of the most important causes of ARDS in humans [11], [12], [13]. Clinical cases and established animal models have revealed that the pathogenesis and pathological features of ARDS induced by different viral pathogens are distinct [14], [15]. However, knowledge of the pathogenesis of 2009 H1N1 virus, especially ARDS induced by 2009 H1N1 virus, is still limited and hinders therapeutic strategies. Therefore, it is necessary to evaluate the pathogenesis of ARDS caused by 2009 H1N1 virus infection in an appropriate animal model to assess potential therapies.

Mice are a good model for evaluating the pathogenesis and antiviral therapy of influenza pneumonia, due to the general fidelity of the illness in mice to the human disease [16]. Moreover, a mouse model of ARDS caused by highly pathogenic H5N1 avian influenza virus infection has been well established [13]. The typical 2009 H1N1 virus, such as A/California/04/2009 (CA/04), can efficiently replicate in mouse lungs without prior host adaptation. However, it only causes moderate lung lesions and no mortality, even when inoculated at a high dose of 10^6^ pfu [17], [18]. Thus, such typical 2009 H1N1 viruses may not be able to induce ARDS in a mouse model.

In the present study, we used a virulent variant 2009 H1N1 virus, which was isolated from a pig and possessed a virulence-associated HA-D222G mutation, to establish an ARDS mouse model. The model established here provides a useful tool to explore the mechanism of ARDS, as well as screening and therapeutic options.

## Results

### Clinical and gross pathologic observation

Six-week-old female mice were infected intranasally (i.n.) with 10^2.5^ pfu SD/09 virus. Some of the infected mice showed signs of illness, such as altered gait, inactivity, ruffled fur, and anorexia on day 2 post-infection (p.i.). From day 2 p.i., the body weight of most mice significantly decreased (Figure S1). By day 6 p.i., most mice presented with more severe clinical signs of respiratory disease, including labored respiration and respiratory distress, and most mice lost almost 20% of their initial body weight. On day 8 p.i., most mice were nearly unable to respond to exterior stimuli, and acute respiratory rates and labored respiration were observed ( Video S1, and Video S2 for control). Approximately 60% of mice died between days 8 and 10 p.i. Gross observation of infected mice showed that the lungs were highly edematous, with profuse areas of hemorrhage and consolidation. No obvious gross lesions were observed in the kidneys, liver, spleen or brain of infected mice.

### Replication kinetics of SD/09 virus in mouse tissues

**Figure 1:**
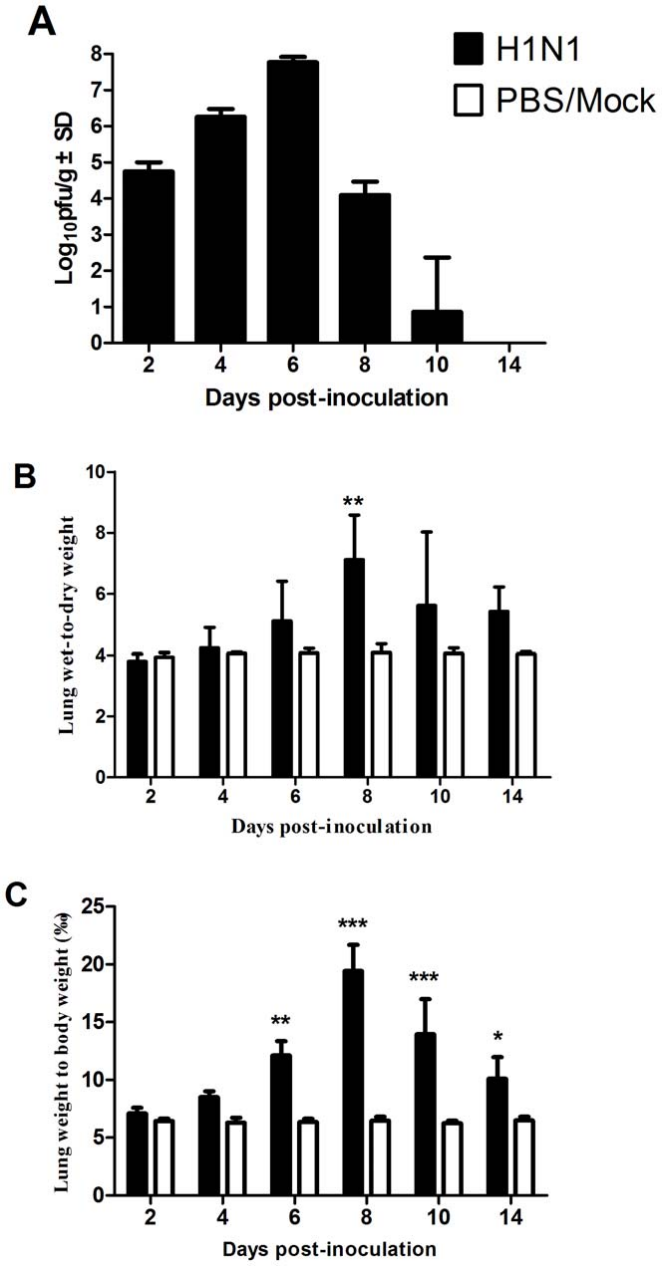
Virus titers in lung (A), wet:dry weight ratio (B) and wet weight∶body weight ratios (C) of SD/09-infected mice. Mice were inoculated i.n. with 10^2.5^ pfu SD/09 viruses; tissues were collected at indicated times p.i. and viruses were titrated in MDCK cells. Body weight, lung wet and dry weight were determined and recorded. The lung wet weight∶body weight ratio and lung wet∶dry weight ratio were calculated and used as an indicator of lung edema. *p<0.05, **p<0.01, ***p<0.001, comparison between ratios obtained from the virus-infected and control groups. Bars represent means ± SD of data from three mice.

Mice were infected i.n. with 10^2.5^ pfu SD/09 virus, and three mice were euthanized on days 2, 4, 6, 8, 10 and 14 p.i., and the virus titers in viscera were determined. As shown in Figure 1A, the virus titer in the lung gradually increased between days 2 and 6 p.i., and reached a peak on day 6 p.i. The virus titers in the lung gradually decreased from day 6 p.i., and only one of three mice possessed detectable virus in the lungs on day 10 p.i. No viruses were detected in other organs, including heart, spleen, liver, kidneys, blood and brain, at the indicated time. These results indicate that SD/09 virus could replicate efficiently in mouse lung but did not cause systemic infection.

### Lung edema induced by SD/09 virus

As shown in Figure 1B, the effect of SD/09 viral infection on lung wet∶dry weight ratio did not change significantly within 2 days p.i. However, a dramatic increase was observed from day 4 p.i., and reached a peak on day 8 p.i., which was nearly twice that observed in control group lungs (p<0.01). The change in lung wet weight∶body weight ratio was similar to the change in lung wet∶dry weight ratio (Figure 1C). The results indicated that the SD/09 virus could induce acute lung edema in mice.

### Histopathology and immunohistochemistry

**Figure 2:**
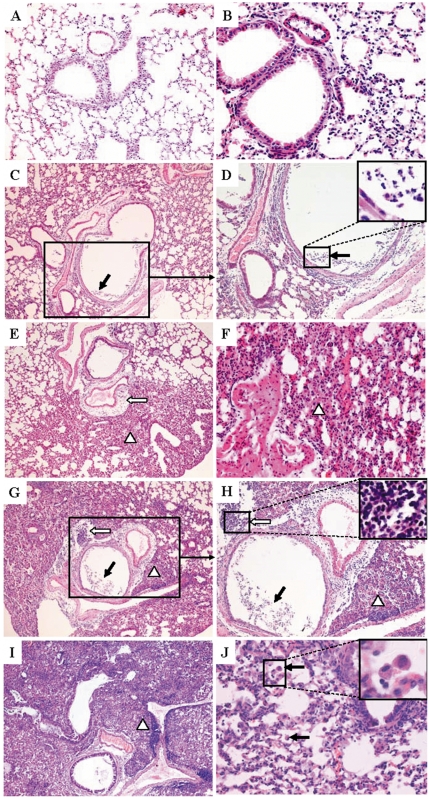
Hematoxylin-eosin stained sections from lung tissues of SD/09-infected mice. Normal mouse lungs (A, B). On day 4 p.i., dropout of mucous epithelium and inflammatory cells adhered to the surface of bronchioles (C, D, solid arrows). On day 6 p.i., severe edema around blood vessels (E, open arrows); dropout of alveolar epithelial, erythrocytes, and inflammatory cells within alveolar spaces (E, F, Δ). On day 8 p.i., the virus caused more severe interstitial pneumonia (G, H, Δ), bronchiolitis (G, H, solid arrows) and peribronchiolitis (G, H, open arrows). On day 14 p.i., the interstitial tissue was thickened with fibrin exudation and organization (I, Δ). Lymphocytes and alveolar macrophages were the predominant inflammatory cells observed (J, solid arrows). Magnification: C, E, G, and I, ×100; A, D and H, ×200; B, F and J, ×400.

**Figure 3:**
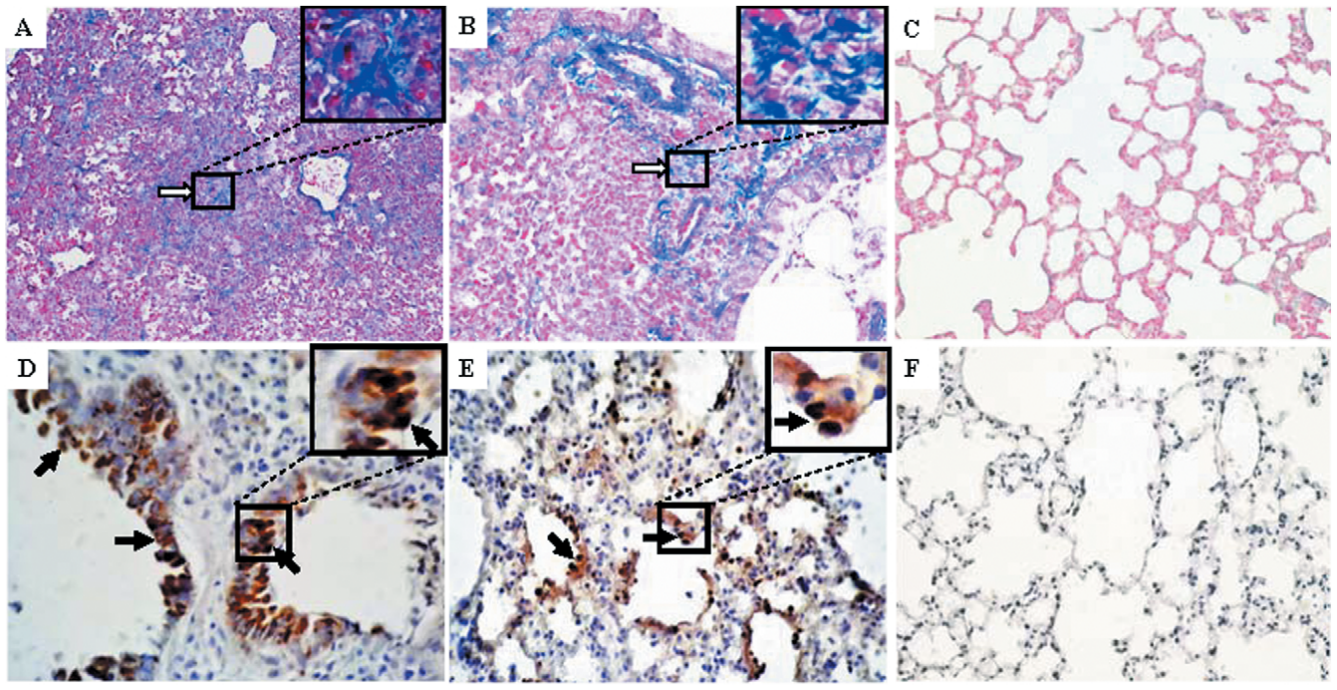
Masson's trichrome stained sections (A–C) and immunohistochemical staining for influenza A nucleoprotein (D–F) of the SD/09-infected mouse lung. Alveoli were collapsed and filled with collagen fibers (A, B, open arrows); normal mouse lungs (C). Influenza A nucleoprotein (brown) in the epithelial cells of the bronchioles (D, solid arrows); viral nucleoprotein in abundant terminal bronchioles epithelial cells and few alveolar epithelial cells (E, solid arrows); normal mouse lungs (F). Magnification: A, ×100; B, C, D, E and F, ×400.

Kinetic observation of lung lesions of SD/09-virus-infected mice is shown in Figure 2. On day 4 p.i., lung lesions were characterized by dropout of mucous epithelium and inflammatory cells adhering to the bronchiolar surface (Figure 2C, D). On day 6 p.i., severe edema could be seen around blood vessels (Figure 2E); interstitial pneumonia was also observed that showed interstitial edema and thickening of the alveolar walls; and the alveolar lumen was flooded with detached alveolar cells, erythrocytes, and inflammatory cells (Figure 2E, F). On day 8 p.i., the virus caused more severe interstitial pneumonia and peribronchiolitis, characterized by edema and extensive of lymphocytes, neutrophils and plasma cells around the area of bronchiolitis (Figure 2G, H). Lesions in the lungs of infected mice were still severe on day 14 p.i., with extensive alveolar collapse, and remaining alveoli were filled with fibrin, desquamated alveolar cells, and inflammatory cells. Lymphocytes and alveolar macrophages were the predominant inflammatory cells observed at high magnification (Figure 2J). Masson's stain revealed that alveolar walls and spaces were filled with collagen fibers (Figure 3A, B), indicating that proliferative fibroblastic lesions may develop. In comparison, lungs from mock-infected control mice had no apparent histological changes (Figure 2A, 2B, 3C).

Immunohistochemistry revealed viral antigens in the epithelial cells of the bronchioles (Figure 3D), terminal bronchioles, and alveolar epithelial cells (Figure 3E). These data indicated that SD/09 virus could infect the epithelia of the lower airway and cause viral pneumonia in mice.

### Arterial blood gas analysis

As shown in Table 1, virus-infected mice showed a slightly decreased partial pressure of arterial oxygen (Pa_O2_), saturation of arterial oxygen (Sa_O2_), and slightly increased partial pressure of arterial carbon dioxide (Pa_CO2_) from days 4 to 6 p.i. Most infected mice presented with severe clinical signs of respiratory distress on day 8 p.i., and blood gas analysis also showed that Pa_O2_ and Sa_O2_ dramatically decreased compared with the controls (p<0.05). These results suggested acute respiratory dysfunction and severe hypoxemia in virus-infected mice.

**Table 1:** Arterial blood gas analysis.

Time (d)	PaO_2_ (mmHg)		PaCO_2_ (mmHg)		SaO_2_ (%)	
	H1N1	Control	H1N1	Control	H1N1	Control
4	82.00±1.41	93.00±9.65	35.00±5.65	29.50±3.54	91.00±1.41	91.50±2.12
6	74.50±2.12	91.00±1.41	41.00±5.65	30.75±3.18	86.50±2.12	91.00±2.83
8	57.50±3.54*	90.50±3.54	52.50±4.95*	28.50±2.83	78.50±3.54**	91.50±0.71
10	73.00±12.72	89.50±2.12	43.00±4.24	29.25±2.47	83.50±3.54	89.00±4.24
14	71.50±12.02	91.50±3.54	39.50±13.44	31.50±6.36	85.50±3.54	91.50±2.12

Mice were inoculated i.n. with 10^2.5^ pfu SD/09 viruses. Arterial blood samples were collected at indicated times p.i.

Values are expressed as mean ± SD.

*p<0.05,

**p<0.01, comparison between virus-infected and control groups.

### Leukocyte counts in Bronchial Alveolar Lavage Fluid (BALF)

The number of leukocytes in BALF from SD/09-infected mice showed an increase from day 4 p.i. (Table 2). The BALF of virus-infected mice on day 6 p.i contained 1.1×10^6^ cells/ml and was significantly different from the 1.6×10^5^ cells/ml observed for PBS-inoculated mice (p<0.001). These data indicate a dramatic increase in inflammatory cells in the lungs of SD/09-infected mice. To quantify the immune cell subpopulations responding to viral infection, we next determined cell differential counts in the infected lungs by Wright staining. Compared with PBS-inoculated animals, mice infected with SD/09 virus exhibited an increase of neutrophils from 4 days p.i., and the peak was 18-fold greater than that of the control group on day 8 p.i.

**Table 2:** White blood cell (WBC) summary and differential counts in BALF.

Time (d)		WBC Summary (1×10^5^)	WBC count %		
			Macrophages	Lymphocytes	Neutrophils
4	H1N1	3.92±0.14	55.16±6.80*	38.26±5.60*	3.69±0.89**
	Control	1.57±0.26	89.38±6.21	8.62±1.25	0.68±0.21
6	H1N1	10.75±5.62***	31.66±2.39**	49.44±6.26**	12.72±2.77***
	Control	1.49±0.29	90.65±2.71	6.97±2.25	0.69±0.35
8	H1N1	9.16±2.21**	26.63±2.19**	40.4±3.22**	28.93±1.98***
	Control	1.85±0.59	88.66±4.34	7.65±1.20	1.58±0.48
10	H1N1	8.59±2.81**	33.23±2.70**	42.25±4.42**	21.62±7.90***
	Control	1.69±0.48	89.32±4.8	6.93±1.2	0.99±0.37

Mice were inoculated i.n. with 10^2.5^ pfu SD/09 viruses, and BALF was prepared at indicated times p.i.

Values are expressed as mean ± SD.

*p<0.05.

**, p<0.01,

***p<0.001, comparison between virus-infected and control groups.

### Peripheral blood leukocyte counts

**Figure 4:**
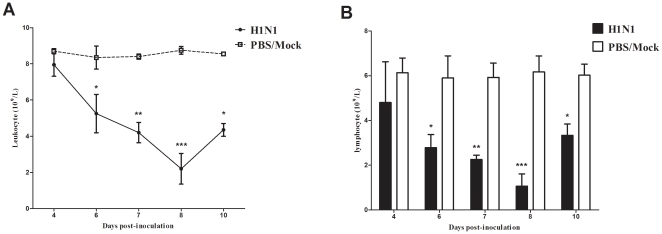
Kinetic analysis of circulating leukocytes (A) and lymphocyte (B) following SD/09 virus infection. Peripheral blood samples were collected at indicated times p.i. The total number of leukocytes and differential blood counts for three individual mice were analyzed. *p<0.05 **, p<0.01, ***p<0.001, comparison between virus-infected and control groups.

Figure 4A shows a progressive reduction in the number of leukocytes observed on days 4–10 p.i. in virus-infected mice. Leukopenia was detected on day 4 p.i., and was statistically significant on day 6 p.i. (p<0.05); the lowest value appeared on day 8 p.i. (p<0.001). Furthermore, differential blood counts revealed that the number of lymphocytes sharply decreased in infected mice. The lowest number of lymphocytes observed occurred on day 8 p.i. (Figure 4B), which dropped to <20% of the control group number (p<0.001).

### Cytokine production following SD/09 infection

**Figure 5:**
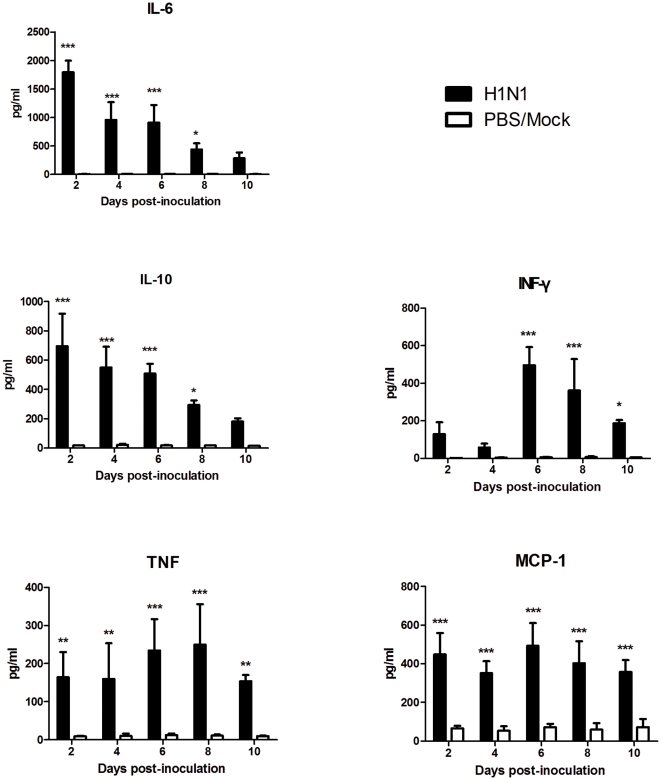
Cytokine levels in virus-infected mouse lungs. Mice were euthanized at indicated times p.i. The collected lungs were weighed, and 10% homogenates were prepared in cold PBS. Cytokine levels from lung homogenates were measured using the Cytokine Bead Array system. *p<0.05, **p<0.01, ***p<0.001, comparison between virus-infected and control groups.

To determine the cytokine responses that occur after SD/09 virus infection, we measured the levels of five cytokines and chemokines in lungs of infected mice on days 2, 4, 6, 8 and 10 p.i. As shown in Figure 5, all five were significantly different between virus-infected and control mice. Interleukin (IL)-6 and IL-10 in the virus-infected mice reached peak levels as early as day 2 p.i. and were significantly higher than those of the control group (p<0.001). Interferon (IFN)-γ, monocyte chemotactic protein (MCP)-1, and tumor necrosis factor (TNF) dramatically increased in mouse lungs on days 6–8 p.i. (p<0.001), consistent with the appearance of pulmonary lesions. These results showed that infection with SD/09 viruses resulted in elevated amounts of proinflammatory chemokines and cytokines in the lungs of mice.

## Discussion

In the spring of 2009, a novel influenza A(H1N1) virus rapidly spread worldwide, resulting in the first influenza pandemic of the 21st century [1]. Critically ill cases caused by 2009 H1N1 virus retrospectively showed that most had progressed or died due to ARDS [4], [6]. However, the pathogenesis and therapeutic intervention of ARDS caused by 2009 H1N1 infection have still not been elucidated. Animal models of disease are important for characterizing pathogenesis and developing the preclinical evidence for revised approaches to ventilating patients with ARDS [19]. Here, we present a mouse model for the study of ARDS induced by SD/09 virus, a virulent 2009 H1N1 variant.

Previous studies have indicated that typical 2009 H1N1 viruses such as CA/04 bind only to α-2,6-linked sialic acid (SA) receptor [17], but only the α-2,3-linked SA receptor is found in the mouse respiratory tract [20]. Therefore, such a typical 2009 H1N1 virus may not be able to induce ARDS in mice. In fact, we used CA/04 virus to induce ARDS in mice; however, animals inoculated with a high dose of CA/04 virus (10^6.5^ pfu) only showed moderate respiratory symptoms, and no lethality was observed (unpublished data). It has been shown that 2009 H1N1 virus possessing a D222G mutation in hemagglutinin (HA) could increase the pathogenicity in mice [21], [22] and binding to the α-2,3 SA receptor [23]. Moreover, clinical data indicate that such variants are only associated with severe H1N1 human infection [24]. Therefore, we suggest that the variant possessing the D222G mutation in HA can induce ARDS in a mouse model. The virus used in the present study was isolated from swine in 2009, and sequence analysis revealed that all the eight genes of the isolate had a close relationship with the 2009 H1N1 influenza virus circulating in humans. Notably, the swine isolate, SD/09, had a D222G mutation in HA. Compared with CA/04 virus (LD_50_>10^6^ pfu), SD/09 showed significantly increased virulence in mice, with an LD_50_ of 10^2.25^ pfu, which was nearly identical to that of the mouse-adapted strain A/Hong Kong/415742Md/09 (LD_50_ = 10^2.2^pfu) [17], [21]. Mice infected i.n. with 10^2.5^ pfu SD/09 virus showed obvious respiratory symptoms, including visually prominent signs of respiratory distress and abdominal respiration, with approximately 60% mortality between days 8 and 10 p.i. The lungs of virus-infected mice were highly edematous, which was also demonstrated by dramatically increased lung wet∶dry weight ratio. Pathological changes presented a progressive pattern, typically diffuse alveolar damage, interstitial and alveolar edema, neutrophil and macrophage-dominant inflammatory cellular infiltration, and areas of hemorrhage and necrotizing bronchiolitis. Arterial blood gas saturation is a key parameter of ARDS in humans [25]. In the present mouse model, Pao_2_ and Sao_2_ of infected mice were significantly lower than in the control group from day 2 p.i., especially on day 8 p.i., where these parameters sharply decreased, and most virus-infected mice began to die. These changes in arterial blood gas demonstrated that most infected mice developed severe hypoxemia consistent with of the appearance of clinical signs and lung lesions of ARDS.

Previous studies showed that mice infected with typical 2009 H1N1 virus only exhibited mild interstitial inflammatory infiltration and limited alveolitis [18], [21], whereas severe lung damage was found in the SD/09-infected mice, including severe edema around the blood vessels and bronchiolitis, and extensive inflammatory accumulation from 4 to 8 days p.i. At 10 days p.i., the surviving mice developed an irreversible fibrosis involving collagen deposition in alveolar walls and spaces, which was similar to that observed in human ARDS patients with 2009 H1N1 infection [26]. Our histopathological results were consistent with ARDS induced by other influenza viruses. Mice infected with mouse-adapted virus of the A/Puerto Rico/8/34 (H1N1), or high pathogenic H5N1 virus also showed a progressive series of pathological changes from interstitial pneumonia to diffuse alveolar damage [13], [27]. However, in contrast to highly pathogenic H5N1 virus, mice infected with SD/09 virus did not show viral spread to extrapulmonary organs.

Immunohistochemical examination revealed the presence of viral antigens in the bronchioles, terminal bronchiolar epithelium, and alveolar epithelial cells. Perhaps SD/09 virus infection of the alveoli, particularly type II pneumocytes, rather than bronchioles, is a key to the development of ARDS. Type II pneumocytes are responsible for the production and secretion of surfactant to lower the surface tension of water and allow membrane separation, and insufficient pulmonary surfactant in the alveoli may result in alveolar collapse [28], [29]. The 1918 pandemic H1N1 and high pathogenic H5N1 viruses preferentially infect type II pneumocytes and alveolar macrophage in mice [15], [30]. Alveolar macrophages may play a critical role in disease pathogenesis, not through production of infectious virus but rather through the upregulation of proinflammatory cytokines that may further damage alveolar pneumocytes [31]. These phenomena suggest that viral cell tropism may determine the processes of ARDS.

Pulmonary aberrant immune response is considered a significant feature of ARDS induced by 2009 H1N1 virus [19], [32]. In the present mouse model, the number of leukocytes observed in the BALF of virus-infected mice significantly increased compared with the control mice on day 8 p.i. Different counts in BALF showed that the proportion of neutrophils dramatically increased. These innate immune cells were capable of reducing the virus load in the lung [33]; however, they could cause lung injury through direct or indirect mechanisms. Neutrophil oxidants and proteases can cause direct injury of cells in the alveolar-capillary membrane [34]. Neutrophils and macrophages can secrete copious amounts of chemokines and cytokines that can recruit more immune cells into lung tissues, and produce a “cytokine storm”, one of the most important factors in the production of ARDS [35].

A retrospective cohort study of 74 2009 H1N1 patients found that higher levels of proinflammatory cytokines and chemokines in plasma were observed in the ARDS-death group compared with the survived-without-ARDS or the mild-disease groups [5]. Another study in critically ill patients with ARDS caused by 2009 H1N1 virus infection has shown that the hallmarks of disease severity were elevated levels of IL-6, IL-15, IL-8 and TNF-α [36]. We examined the levels of five cytokines and chemokines in infected mouse lungs and found significant differences between the virus-infected and mock groups. It has proved that high levels of IL-6 were able to mediate acute lung injury [37], and had a negative correlation with the Pa_O2_∶Fi_O2_ ratio in severely affected patients with 2009 H1N1 virus infection [36]. Our data showed SD/09 viral infection induced high levels of IL-6 in mouse lung, which may also play an important role in the course of ARDS. Hagau etc. found the levels of TNF-α increased significantly in the 2009 H1N1-related ARDS patients [36]. In present study, TNF levels also dramatically increased in the lungs of virus-infected mice, and were consistent with the clinical symptoms and reached peak levels when mice began to die. In addition, high levels of IL-10, IFN-γ and MCP-1 were also present in the virus-infected mouse lungs, similar to observations found in severely affected humans with 2009 H1N1 infection [38].

In summary, we successfully established an ARDS mouse model induced by a virulent 2009 H1N1 variant, which demonstrated key human ARDS clinical and pathological features, such as respiratory distress, low Pa_O2_, exudative, proliferative and fibrotic lung, and high levels of inflammatory cells and cytokines. The mouse model may contribute to the study of the pathogenesis and therapy of ARDS induced by 2009 H1N1 virus.

## Materials and Methods

### Ethics statement

All animal research was approved by the Beijing Association for Science and Technology (approval ID SYXK (Beijing) 2007-0023) and complied with the guidelines of Beijing Laboratory Animal Welfare and Ethics of the Beijing Administration Committee of Laboratory Animals.

### Viruses

The pandemic 2009 H1N1 virus, A/swine/Shandong/731/2009 (SD/09), was isolated from swine in Shandong Province, China, in December 2009. The complete genomic sequences for this virus are available in the GenBank database under accession numbers FJ951848–FJ951855. All the eight gene segments from the swine isolate have a close relationship to 2009 H1N1 influenza virus circulating in the human population and possess homologies of 99.3–99.9% to those of a human representative 2009 H1N1 strain A/California/04/2009. Virus stock was propagated in the Madin–Darby canine kidney (MDCK) cells at 37°C for 48 h and stored at −80°C for use in all the experiments. The titers of stock viruses were determined by plaque assays in MDCK cells. To determine LD_50_ of SD/09 virus, eight 6-week-old female BALB/c mice per group were inoculated i.n. with 10^1^–10^6^ pfu (50 µl) viruses and monitored for 14 days. The value of MLD50 was calculated using the Spearman–Karber method and expressed by pfu per MLD_50_ [39].

### Experimental infections

We evaluated the pathogenicity of the virus in mice and found that it could efficiently replicate in the lungs of mice with high lethality (10^2.25^ pfu per MLD_50_). To determine the optimal dose of inoculation, 10 mice in each group were infected i.n. with 10^1.5^, 10^2.5^ or 10^3.5^ pfu viruses, and the signs, body weight, and mortality were monitored daily for each group for 14 days. Pilot experiments indicated that a dose of 10^2.5^ pfu was optimal, because the course of the disease was prolonged and the mice presented with obvious signs of respiratory illness.

BALB/c mice were lightly anesthetized and inoculated i.n. with 50 µl 10^2.5^ pfu SD/09 virus in PBS. Mock-infected animals were inoculated i.n. with 50 µl PBS. At the indicated time, infected mice were sacrificed, and the parameters that present the course of the disease were determined. Twenty mice (10 infected with SD/09 virus and 10 inoculated with PBS) were used to investigate clinical signs and mortality for 14 days.

### Virus titration

Three mice were euthanized on days 2, 4, 6, 8, 10 and 14 p.i. and their organs were collected. The collected tissues were weighed, and 10% homogenates were prepared in cold PBS. The homogenates were centrifuged at 3000 rpm for 10 min to remove cell debris, and then the supernatants were 10-fold serially diluted for viral titer determination by plaque assay in MDCK cells. Virus titers were expressed as mean log pfu/g ± standard deviation (SD).

### Assessment of lung water content

Three mice were euthanized on days 2, 4, 6, 8, 10 and 14 p.i., and the lungs were removed and weighed and then desiccated in an oven at 60°C for 72 h. The lung wet weight∶body weight ratio and lung wet∶dry weight ratio were calculated and used as an indicator of lung edema, as previously described [40].

### Histopathological examination

Three mice were euthanized on days 4, 6, 8 and 14 p.i. The lungs were fixed in 10% buffered formalin, embedded in paraffin, sectioned, and stained with hematoxylin and eosin. Lungs on day 14 p.i. were also stained with Masson's trichrome.

### Immunohistochemistry

Lung tissue sections taken on day 6 p.i. were stained for influenza A virus antigens. An anti-influenza nucleoprotein monoclonal antibody (AA5H; Abcam, Hong Kong) was used to identify influenza A virus nucleoprotein in sections. Secondary antibody (Millipore, Billerica, MA, USA) against the primary antibody was labeled with horseradish peroxidase, and the color reaction was developed with a horseradish peroxidase reaction kit (diaminobenzidine- tetrahydrochloride; Sigma, St. Louis, MO, USA).

### Arterial blood gas analysis

Blood gas analysis was performed as previously described [13], [41]. Three mice were anesthetized with Zoletil (tiletamine-zolazepam; Virbac; 20 µg/g) on days 4, 6, 8, 10 and 14 p.i. Arterial blood samples were withdrawn into a heparinized syringe by percutaneous left ventricular sampling of lightly anesthetized mice that were spontaneously breathing room air. Blood gas analysis was immediately performed using a Vetstat Electrolyte and Blood Gas Analyzer (Idexx laboratories, Westbrook, MA, USA).

### Leukocyte counts in BALF

Leukocyte counts in BALF were performed as previously described [42], [43]. Briefly, three mice were euthanized on days 4, 6, 8 and 10 p.i., and the lungs were lavaged twice *in situ* with the chest cavity opened by midline incision with a total volume of 1.0 ml saline (4°C) inserted through an endotracheal tube. The rate of recovery of BALF was not less than 90% for all animals tested. After the amount of fluid recovered was recorded, an aliquot of BALF was diluted 1∶1 with 0.01% crystal violet and 2.7% acetic acid for leukocyte staining and erythrocyte hemolysis. The number of leukocytes in the BALF was counted with a hemacytometer under a microscope. For differential counts, the BALF samples from each mouse were stained with Wright stain, and the numbers of monocytes, neutrophil and lymphocytes were determined, on the basis of morphologic criteria, under a light microscope, with evaluation of at least 200 cells per slide. All slides were counted twice by different observers blinded to the status of the animal.

### Peripheral blood leukocyte counts

Heparinized blood samples were collected on days 4, 6, 8 and 10 p.i. The total numbers of leukocytes and differential blood counts for three individual mice were analyzed using an automated hematology analyzer.

### Cytokine analysis in mouse lung

IL-6, IL-10, TNF, IFN-γ and MCP-1 levels were determined in lung homogenates using a cytometric bead array technique (BD Cytometric BEAD Array Mouse Inflammation Kit; BD Bioscience, San Diego, CA, USA) according to the manufacturer's instructions. Briefly, 50 µl mouse inflammation capture bead suspension and 50 µl PE detection reagent were added to an equal amount of sample standard dilution and incubated for 2 h at room temperature in the dark. Subsequently, samples were washed by adding 1 ml wash buffer and centrifugation at 200× g at room temperature for 5 min. Supernatants were discarded and 300 µl wash buffer was added. Samples were analyzed on a BD FACSArray bioanalyzer (BD Bioscience) according to the manufacturer's instructions. Standard curves were prepared similar to the method above. Data were analyzed using BD CBA Software (BD Bioscience). Finally, the chemokine or cytokine levels were recorded as pg/ml homogenate.

### Statistics

Data were analyzed by two-way analysis of variance using GraphPad Prism version 5.00 (GraphPad Software, San Diego, CA, USA). When a significant effect was observed, pairwise comparisons were performed using the Bonferroni post-hoc test. All data are reported as mean ± SD.

## Supporting Information

Figure S1 **Body weight changes p.i.** Mice were inoculated i.n. with indicated doses of SD/09 viruses or PBS. Body weights were recorded every day p.i. The percentage of the body weight p.i. to the initial weight is shown. Bars represent means ± SD.
(TIF)

Video S1 **Clinical symptoms of the mice infected with SD/09 virus on day 8 p.i.**
(MP4)

Video S2 **Normal appearance of the mice inoculated with PBS on day 8 p.i.**
(MP4)
